# 18S rRNA metabarcoding diet analysis of a predatory fish community across seasonal changes in prey availability

**DOI:** 10.1002/ece3.4857

**Published:** 2019-01-10

**Authors:** Justin M. Waraniak, Terence L. Marsh, Kim T. Scribner

**Affiliations:** ^1^ Department of Fisheries and Wildlife Michigan State University East Lansing Michigan; ^2^ Department of Microbiology and Molecular Genetics Michigan State University East Lansing Michigan; ^3^ Department of Integrative Biology Michigan State University East Lansing Michigan

**Keywords:** diet composition, dietary overlap, freshwater fish, larval fishes, macroinvertebrates, temporal variability

## Abstract

Predator–prey relationships are important ecological interactions, affecting biotic community composition and energy flow through a system, and are of interest to ecologists and managers. Morphological diet analysis has been the primary method used to quantify the diets of predators, but emerging molecular techniques using genetic data can provide more accurate estimates of relative diet composition. This study used sequences from the 18S V9 rRNA barcoding region to identify prey items in the gastrointestinal (GI) tracts of predatory fishes. Predator GI samples were taken from the Black River, Cheboygan Co., MI, USA (*n* = 367 samples, 12 predator species) during periods of high prey availability, including the larval stage of regionally threatened lake sturgeon (*Acipenser fulvescens *Rafinesque 1817) in late May/early June of 2015 and of relatively lower prey availability in early July of 2015. DNA was extracted and sequenced from 355 samples (96.7%), and prey DNA was identified in 286 of the 355 samples (80.6%). Prey were grouped into 33 ecologically significant taxonomic groups based on the lowest taxonomic level sequences that could be identified using sequences available on GenBank. Changes in the makeup of diet composition, dietary overlap, and predator preference were analyzed comparing the periods of high and low prey abundance. Some predator species exhibited significant seasonal changes in diet composition. Dietary overlap was slightly but significantly higher during the period of high prey abundance; however, there was little change in predator preference. This suggests that change in prey availability was the driving factor in changing predator diet composition and dietary overlap. This study demonstrates the utility of molecular diet analysis and how temporal variability in community composition adds complexity to predator–prey interactions.

## 1 INTRODUCTION

Characterization of predator diets and food web interactions is important to the understanding of community functioning and management of freshwater systems (Thompson, Dunne, & Woodward, 2012; Vaughn, 2010).

Quantifying the dietary composition of predator fishes in the context of relative prey availability in the environment is necessary to investigate the ecological relationships between predators and their prey and to determine predator–prey preference. Traditionally, diet analyses have been conducted through morphological identification of prey collected from predator gastrointestinal contents, but identification of diet contents using these methods is often inaccurate (Buckland, Baker, Loneragan, & Sheaves, 2017; Schooley et al., 2008). More recent molecular methods have been applied to overcome some of these shortcomings (Berry et al., 2015; Carreon‐Martinez, Johnson, Ludsin, & Heath, 2011; Sheppard & Hardwood, 2005). Application of a molecular approach to quantify diet compositions of multiple species in a predator community, combined with data on prey resource availability, can lead to greater understanding of possible competitive interactions between predators as well as how changes in prey abundance affect these interactions.

Predator preferences and dietary overlap among predators are driven in part by the prey relative abundance. High abundance of prey leads to higher encounter rates and in some cases can cause abundant prey taxa to be targeted by predators (i.e., positive frequency dependence; Ims, 1990; Murdoch, 1969). High abundance of prey can reduce interspecific competition, allowing predators to coexist despite high dietary overlap (Gray, Boltz, Kellogg, & Stauffer, 1997; Kelling, Isermann, Sloss, & Turnquist, 2016; Michaletz, 1997). Seasonal fluctuations in prey abundance and species composition are common features of riverine communities (Brown & Armstrong, 1985; Gray et al., 1997; Smith & King, 2005). Synchronized emergence and dispersal of larval fishes and aquatic macroinvertebrates may be an adaptive strategy to swamp predators, leading to periods where foraging predators are saturated by prey (Frank & Leggett, 1983; Ims, 1990). The seasonal influx of prey, comprised of the early life stages of river spawning fishes and emergence of certain aquatic macroinvertebrate taxa, can alter trophic interactions between predators and prey, and change the diet composition and dietary overlap of the predatory fishes in rivers. Understanding how the variation in the prey community affects these relationships is important to conservation, as predator preference can indicate what members of the community act as important energetic links between trophic levels (Chesson, 1978; Ivlev, 1961), and estimates of diet similarity between two predator species may indicate the degree of interspecific resource competition (Schoener, 1970).

DNA‐based molecular methods are useful tools for analyzing the diets of fishes in freshwater food webs with greater accuracy and resolution than traditional morphology‐based methods (Carreon‐Martinez & Heath, 2010; Pompanaon et al., 2012). Metabarcoding is one molecular technique, utilizing conserved regions of DNA to amplify sequences in samples that are unique in different taxa (King, Read, Traughott, & Symondson, 2008). Molecular techniques have advantages over morphological analyses of diets that require visual identification of partially digested prey items. Molecular barcoding is capable of identifying prey items to a greater taxonomic resolution and for longer periods after consumption (Berry et al., 2015; Carreon‐Martinez et al., 2011; Schooley et al., 2008; Sheppard & Hardwood, 2005). The greater diet breadth and taxonomic resolution that can be achieved through metabarcoding diet analysis can allow characterization of the degree of niche partitioning among species, revealing how predators can partition resources to reduce interspecific competition (Albaina, Aguirre, Abad, Santos, & Estonba, 2016; Katzinel et al., 2015; Leray, Meyer, & Mills, 2015). Dietary overlap estimated by molecular methods could also be significantly different than nonmolecular studies estimated depending on the prevalence of soft‐bodied prey items in predator diets (Gebremedhin et al., 2016; Soininen et al., 2015), which are often difficult to detect in morphological diet studies due to rapid digestion times (Carreon‐Martinez et al., 2011; Ley et al., 2014).

In temperate streams in northern Michigan, USA, where this study was conducted, the period of high prey dispersal in the drift (mid‐May to early June) is predominated by the emergence of larval suckers (Family: Catostomidae) and larval lake sturgeon (*Acipenser fulvescens *Rafinesque 1817), a species of conservation concern (Auer & Baker, 2002; Smith & King, 2005). This period also coincides with the emergence of several aquatic insects, including families Heptageniidae, Isonychiidae, and Perlidae (Scribner, unpublished data). This study examined associations between abundance of prey in the drift and the diet composition of predators that prey upon larval lake sturgeon. The goals of this research were to (a) characterize the diets of predatory fish during and after the high prey biomass drift period using metabarcoding molecular diet analysis, (b) measure dietary overlap between predator species during and after the drift period, and (c) quantify predator diet preferences and changes in preference using metabarcoding diet data combined with composition estimates from stream surveys of the prey community.

## 2 MATERIALS AND METHODS

### 2.1 Study area and sample collection

Sampling was conducted in the Upper Black River (UBR; Cheboygan County, MI, USA), the largest tributary of Black Lake, a 4,100 ha inland lake in the northern lower peninsula of Michigan. Black Lake supports a population of ~1,200 adult lake sturgeon (Pledger, Baker, & Scribner, 2013), which spawn solely in the UBR. Larval lake sturgeon disperse from the UBR in late spring, often coinciding with the outmigration of larval white suckers [*Catostomus commersonii *(Lacepède, 1803)] and silver redhorse [*Moxostoma anisurum* (Rafinesque, 1820)], and the emergence of several species of aquatic insects (e.g., Families: Heptageniidae, Isonychiidae, Perlidae), leading to a high abundance and diversity of available prey for predatory fishes in the system. This high prey abundance contrasts with the comparatively lower abundance of available prey present in the drift by mid‐summer in the UBR.

**Figure 1:**
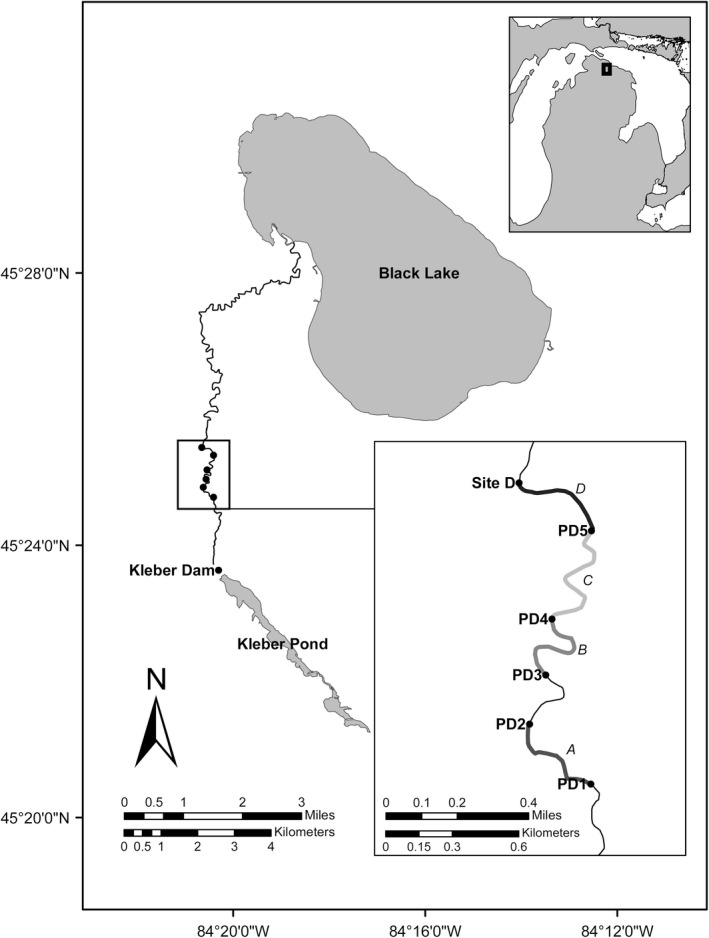
Map of the study area highlighting the D‐frame drift net survey sites of the prey community (black points; PD1, PD3, PD4, and PD5) and the 0.5 km predator electrofishing transects (bold gray lines; A, B, C, and D) in the upper Black River, Cheboygan County, MI. Transects A and B were characterized by gravel substrate and transects C and D were characterized by sand

**Figure 2:**
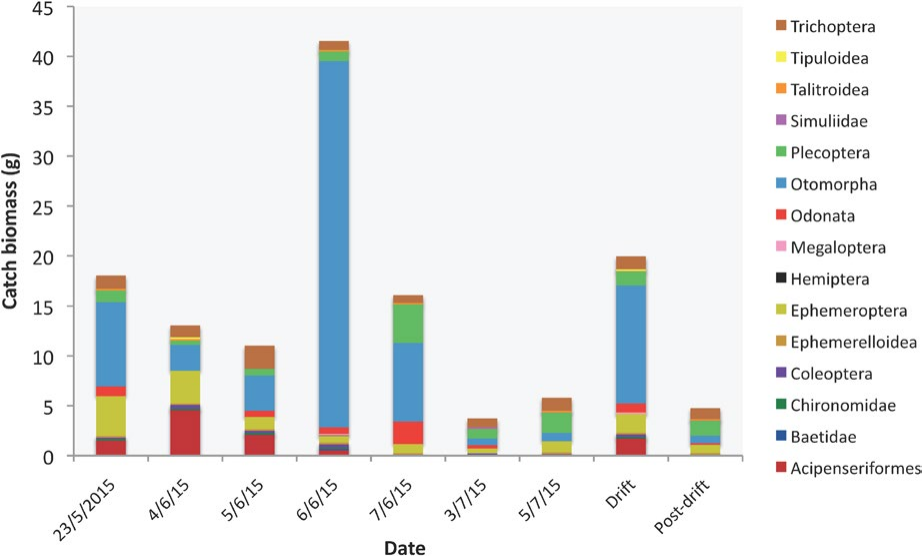
Estimated catch dry weight biomass (g) for aquatic macroinvertebrate and larval fish ecologically significant units (ESUs) observed during each night of the survey during the drift (23 May to 7 June) and postdrift periods (3 July to 5 July), and averages for each period

Sampling of drifting prey was conducted during 2015 at four sites downstream of lake sturgeon spawning sites. Two sites consisted of predominately habitats composed of gravel substrate (Figure 1; PD1 and PD3), and two sites further downstream were located in habitats composed predominately of sand substrate (Figure 1; PD4 and PD5). Sampling dates were divided into two periods. The first period, “drift,” occurred when larval lake sturgeon and catostomids were observed in survey samples. “Drift” samples were collected for five days during the lake sturgeon and catostomids drift period in 2015 (24 May, 4–7 June). The second period, “postdrift,” occurred when larval lake sturgeon and catostomids were no longer observed in the survey samples. The “postdrift” period began 2 days after no larval lake sturgeon or catostomids were observed in the drift surveys and included drift sampling on two nights (3 July, 5 July). The abundance of drifting larval lake sturgeon and co‐distributed larval fish and macroinvertebrate prey taxa was quantified using D‐frame drift nets (Auer & Baker, 2002). Beginning at 21:00, five D‐frame drift nets with 1,600 µm mesh and detachable cod ends were set at one of the sampling sites each night. To estimate the proportion of the river sampled by the drift nets, total river discharge (m^3^/s) and the discharge entering nets were measured using a Marsh McBurney Flow‐Mate 2000 (Hach Company, Loveland, CO, USA). Contents of the cod ends were collected hourly between 22:00 and 02:00. Larval lake sturgeon were counted on site and returned to the river. 5% subsamples of the cod end contents were collected for each hour and preserved in 95% ethanol. Sucker larvae and invertebrates in the preserved samples were later counted and macroinvertebrate larvae were morphologically identified to the family level. Dry weight biomass estimates for individual fish and aquatic insect larvae were collected for most families observed during drift sampling (Table 1). These estimates or the estimate from a closely related family were used to estimate total nightly catch biomass by multiplying the nightly catch counts by the individual dry weight biomass for each taxon and adjusting for subsample size (Figure 2).

**Table 1:** Dry weight biomass (g) estimates for individual prey for each family represented in the D‐frame drift net surveys and the estimated catch biomass of each prey family for each night. Some prey families were grouped together under the same ecologically significant unit (ESU), indicated in parentheses after the family name

	Prey families										
	Catostomidae	Acipenseridae	Heptageniidae (ESU—Other Ephemeroptera)	Baetidae	Ephemerellidae (ESU—Ephemerelloidea)	Isonychiidae (ESU—Other Ephemeroptera)	Siphlonuridae (ESU—Other Ephemeroptera)	Ephemeridae (ESU—Other Ephemeroptera)	Brachycentridae (ESU—Trichoptera)	Glossosomatidae (ESU—Trichoptera)	Helicopsychidae (ESU—Trichoptera)
Individual dry mass (mg)	1.193	8.5	1.768	0.366	1.255	4.145	6.829	5.578	1.458	6.948	0.914
23/5/15 estimated catch biomass (g)	8.42	1.65	1.03	0	0.15	2.98	0	0	0	0	0.02
4/6/15 estimated catch biomass (g)	1.89	4.71	0.74	0.02	0.18	2.98	0	0	0	0	0
5/6/15 estimated catch biomass (g)	3.53	2.21	0.57	0.02	0.08	0.50	0.14	0	0	0.14	0.53
6/6/15 estimated catch biomass (g)	36.70	0.66	0.46	0.05	0.18	0.08	0.14	0.11	0	0.14	0.02
7/6/15 estimated catch biomass (g)	7.90	0.12	0.57	0.01	0.13	0.33	0	0	0.03	0.14	0
3/7/15 estimated catch biomass (g)	0.67	0	0.18	0.14	0	0.33	0	0	0.26	0	0
5/7/15 estimated catch biomass (g)	0.84	0	0.53	0.07	0.03	0.58	0	0	0.12	0	0
Average catch biomass “drift” (g)	11.69	1.87	0.67	0.02	0.14	1.38	0.05	0.02	0.01	0.08	0.11
Average catch biomass “postdrift” (g)	0.75	0	0.35	0.10	0.01	0.46	0	0	0.19	0	0

	Prey families														
	Hydropsychidae (ESU—Trichoptera)	Lepidostomatidae (ESU—Trichoptera)	Leptoceridae (ESU—Trichoptera)	Limnephilidae (ESU—Trichoptera)	Perlidae (ESU—Plecoptera)	Perlodidae (ESU—Plecoptera)	Chironomidae	Simuliidae	Sialidae (ESU—Megaloptera)	Amphipoda (ESU—Talitroidea)	Elmidae (ESU—Coleoptera)	Psephenidae (ESU—Coleoptera)	Cordulegastridae (ESU—Odonata)	Gomphidae (ESU—Odonata)	Total catch biomass (g)
Individual dry mass (mg)	5.92	3.00	0.81	1.27	27.6	5.61	0.15	0.21	3.13	0.59	1.10	3.77	16.0	16.0	—
23/5/15 estimated catch biomass (g)	0.83	0.30	0.18	0	1.10	0.22	0.01	0	0	0.01	0.09	0.08	0	0.96	18.03
4/6/15 estimated catch biomass (g)	1.07	0.18	0.13	0	1.66	0	0.02	0	0	0	0.18	0	0	0.64	14.40
5/6/15 estimated catch biomass (g)	0.83	0.60	0.19	0.03	0.55	0.11	0.04	0	0	0	0.20	0.08	0.32	0.32	10.99
6/6/15 estimated catch biomass (g)	0.47	0.18	0.06	0	1.10	0	0.10	0	0.06	0.01	0.26	0.08	0	0.64	41.50
7/6/15 estimated catch biomass (g)	0.59	0	0.02	0	3.87	0.11	0	0	0	0.02	0.02	0	0	1.92	15.78
3/7/15 estimated catch biomass (g)	0.59	0	0.03	0	1.10	0	0.02	0.01	0	0	0.07	0	0	0.32	3.72
5/7/15 estimated catch biomass (g)	1.07	0	0.11	0	2.21	0	0.02	0.01	0	0.01	0.22	0	0	0	5.82
Average catch biomass “drift” (g)	0.76	0.25	0.12	0.01	1.66	0.09	0.03	0	0.01	0.01	0.15	0.05	0.06	0.90	20.14
Average catch biomass “postdrift” (g)	0.82	0	0.07	0	1.66	0	0.02	0.01	0	0.01	0.14	0	0	0.16	4.75

Electrofishing surveys were conducted the day following drift sampling to collect diet samples of predatory fishes (*n* = 367 samples from 12 predator species). A barge electrofishing unit including a three‐person crew sampled a 0.5 km stream transect directly downstream of the site where drift sampling was conducted the previous night (Figure 1; Transects A, B, C, and D). Electrofishing voltage and amperage were set to 400 V at 4 A, respectively. Two crew members carried anodes and collected fish, and the third crew member moved the barge upstream and stored captured fish in a live well. Predator fish were sacrificed with an overdose of MS222 (0.4 mg/ml). Total length and species of all fish captured during the survey were recorded. Sacrificed fish were placed in Whirl‐Paks (Nasco, Fort Atkinson, WI, USA) and stored in a −20°C freezer within 2 hr. Predators were dissected, the entire GI tracts were removed, and contents were carefully extracted to minimize the amount of predator tissue in the sample. Diet samples were preserved in 95% ethanol and stored at −20°C prior to DNA extraction.

### 2.2 DNA extraction and sequencing

Diet samples were mixed by hand, and pieces of tissue were broken apart with forceps and sterile toothpicks and thoroughly vortexed to homogenize the samples and to ensure representative subsampling. About 50–100 mg of tissue from the GI tract diet samples was used in each DNA extraction and washed with sterile water to remove excess ethanol. This was usually the entire diet sample. DNA was extracted according to a modified version of the QIAamp Stool Mini Kit (QIAGEN, Hilden, Germany) protocol. Lysis in InhibitEx Buffer from the QIAmp Stool Mini Kit was extended to 30 min at 72°C. Samples were also further homogenized with a 10‐min bead‐beating step using 0.70 mm garnet beads (MOBIO, Carlsbad, CA, USA) after lysis buffer and proteinase K were added to the sample. DNA was eluted, and DNA concentration was quantified using an ND‐1000 nanodrop spectrophotometer (NanoDrop Technologies Inc., Wilmington, DE, USA). If the nanodrop spectrophotometer revealed a high concentration of contaminants (260/280 < 1.7) in the sample, a salt precipitation using cold 100% ethanol and 0.15 M sodium acetate was used to clean samples. All samples were diluted using sterile water to a standard concentration of 20 ng/µl of DNA. An empty microcentrifuge tube was used as a negative control for each extraction, and three negative controls were randomly selected for sequencing.

The coding region for 18S V9 rRNA (~200 bp; Stoeck et al., 2010) was amplified with universal eukaryotic primers 1391F (5′‐GTACACACCGCCCGTC‐3′; Lane, 1991) and EukB (5′‐TGATCCTTCTGCAGGTTCACCTAC‐3′; Medlin, Elwood, Stickel, & Sogin, 1988). PCR amplification of the 18S V9 region was carried out in 50 µl reactions using 20 ng of template DNA, 0.5 µmol of each primer, 200 µmol of dNTPs, 5 U of Taq polymerase, and 1X Taq reaction buffer (Invitrogen, Carlsbad, CA, USA). Reactions were amplified starting with an initial 5 min incubation at 95°C, followed by 30 cycles of 94°C for 30 s, 57°C for 45 s, and 72°C for 60 s before a final elongation step of 72°C for 2 min. These primers were chosen for their relatively short target sequence (~200 bp), the large taxonomic breadth encompassed, and because preliminary screening indicated sequences from lake sturgeon, suckers, and all major invertebrate families identified in UBR drift survey samples for the target region were available on GenBank (NCBI). Samples were sent to the Research Technology Support Facility (RTSF) at Michigan State University (East Lansing, MI, USA) for DNA sequencing. Sequencing libraries were created using a two‐step PCR approach, first amplifying the targeted sequences with the 18S V9 primers with CS1 and CS2 tag sequences (Fluidigm, South San Francisco, CA, USA) added to the primers. Second, these amplicons could now be uniquely indexed with sample‐specific barcodes and Illumina adaptor sequences in a subsequent PCR reaction. Indexed amplicons were normalized using a SequalPrep Normalization plate (Invitrogen) and pooled for sequencing. Sequencing was performed using 150 bp paired‐end reads using an Illumina MiSeq v2 flow cell sequencing platform with a 500 cycle v2 reagent cartridge (http://rtsf.natsci.msu.edu/genomics).

### 2.3 DNA sequence processing

Sequences were processed in *mothur* v 1.38 (Schloss et al., 2009). Similar paired‐end reads (<2 bp difference) were merged to generate a list of unique sequences. Sequences were screened for quality by removing sequences that were longer than the target size after primer sequences were trimmed (>175 bp), unique sequences that appeared only once, sequences with homopolymer regions ≥8 bp, and chimera checking. Sequences were clustered into unique OTUs if there were ≤2 bp differences between sequences. To standardize sequence sampling coverage between samples, all samples were rarefied to 1950 sequences. Rarefaction subsamples a consistent number of reads from each sample to standardize each sample to the same number of sequences while still accurately reflecting the relative abundance of each unique sequence present in the sample. Rarefaction curves for all samples were created to ensure the rarefaction did not artificially reduce the number of observed OTUs (Supporting Information Figure S1). Twelve samples with insufficient sequence numbers were discarded from further analysis.

OTUs appearing in the 2,500 most common unique OTU sequences were identified to the lowest taxonomic classification identifiable from matches on GenBank (>95% sequence similarity with 100% sequence coverage), with family being the lowest taxonomic classification used if lower classifications met these criteria. Only bilaterian DNA sequences were considered as potential prey items, as microbial sequences were more likely parasites or incidentally ingested. Prey taxa were divided into ecologically significant units (ESUs) based on the lowest taxonomic level that could be confidently identified. The number of OTU sequences from the same ESU was summed together within each sample. Sequences matching the identity of the predator the GI tract sample was taken from were removed from that sample, but retained in samples from predators of different taxa. Rarefied GI tract diet samples with <20 sequences (1% of sequences) from likely diet items were removed from the dataset. These samples were likely taken from fish with empty or near empty stomachs, so most reads came from their own tissue, ingested environmental DNA, or resident parasites in their GI tract. All remaining samples were standardized so sequences of each diet item were represented as the proportion of all prey sequences in a sample.

### 2.4 Examination of relationship between sequence count and biomass

To test for bias due to differences in copy number of prey rRNA or differential amplification of prey sequences by the 18S rRNA universal primers and to empirically demonstrate the relationship between relative sequence abundance and biomass for a number of prey taxa, a homogenate of invertebrate and fish tissue was created using 16 of the most abundant families collected during the drift survey. Preserved samples were removed from ethanol and air‐dried for 24 hr. Three mixtures of roughly equal biomass from each of family were held at −80°C for 1 hr and mechanically homogenized with a mortar and pestle for 20 min (Table 2). When possible, insect heads and limbs were used to create the homogenate to avoid possible PCR inhibitors and contaminants in digestive tracts. Two 100 mg subsamples were taken from each mixture for DNA extraction using the same process as described previously. DNA sequencing of the drift homogenate was the same as described for the diet samples. Sequences were processed in *mothur* v 1.38 with the same protocol described previously with the only difference being that sample were rarefied to 9,450 sequences (the number of sequences present in the sample with the fewest sequences) instead of 1,950.

**Table 2:** Mean percentages of biomass and sequences and the relative correction factor (RCF) for 15 families used to test differential amplification of the universal primers in homogenized mixed samples of tissue. RCFs >1 indicate a family was overrepresented by sequence abundance and RCFs <1 indicate a family was under‐represented by sequence abundance

Major groups	Family	Biomass (%)	Sequences (%)	RCF
Fish	Acipenseridae	8.68	15.49	1.928
	Catostomidae (Otomorpha)	7.18	3.72	0.499
	Centrarchidae (Perciformes)	6.77	7.00	1.037
Insects	Ephemerellidae	3.10	3.02	0.973
	Isonychiidae/Siphlonuridae/Heptageniidae (Other Ephemeroptera)	21.79	39.80	2.373
	Hydropsychidae/Leptoceridae (Trichoptera)	14.72	8.02	0.505
	Chironomidae	1.03	1.60	1.548
	Elmidae	7.30	0.96	0.123
	Perlidae	7.49	0.87	0.108
	Gomphidae	8.88	0.09	0.009
Crustaceans	Cambaridae	6.42	19.34	3.495

Relative correction factors (RCFs) were calculated for each family based on the relative abundance of sequences compared to the relative biomass in the samples (Thomas, Deagle, Eveson, Harsch, & Trites, 2016; Equation (1).

(1) $${\text{RCF}}_{\text{t}}=S_{\text{t}}S_{\text{m}}^{-1}\times B_{\text{m}}B_{\text{t}}^{-1}$$

where RCF_t_ is the relative correction factor for the target family (t), *S*_t_ is the sequence proportion for the target family in a sample, *S*_m _is the sequence proportions for other families in a sample, *B*_t_ is the biomass proportion of the target family in a sample, and *B*_m _is the biomass proportion of other families in a sample. RCFs >1 indicate a family was overrepresented by sequence abundance and RCFs <1 indicate a family was under‐represented by sequence abundance.

### 2.5 Statistical analysis

Multivariate analyses were conducted in R Statistical Software v. 3.2.2 (R Core Team, 2015) using the vegan library (Oksanen et al., 2016). A principal coordinates analysis (PCoA) was conducted on the proportions of diet items in each diet sample using Bray–Curtis distances. Correlations between the original matrix of diet item proportions and the eigenvectors of the first two principal coordinates were calculated to analyze which prey items were explaining most of the variation in the diet. The first two principal coordinates were also plotted by predator species and by time period collected with 80% confidence intervals around each category.

To test the effects of predator species, sampling period, and substrate on the diet composition among members of the predator community, a PERMANOVA analysis was performed on the Bray–Curtis distance matrix of the diet proportions using the adonis function (Oksanen et al., 2016). Each PERMANOVA was run with 1,000 permutations. Predator species, sampling period, and substrate were all treated as fixed effects and all interactions among the fixed effects were analyzed. If the three‐way interaction was not significant, the interaction was removed and the model was fit again. The model was fit again if none of the two‐way interactions between fixed effects was significant. If an interaction was significant, separate PERMANOVAs were performed on the data from each level of one the interacting factors, testing the effect of the other interacting factor.

Dietary overlap between each species was calculated for both the drift and postdrift sampling periods. The diets from fish predators of the same species and sampled during the same time period (drift or postdrift) were pooled together, proportions of each prey ESUs were calculated in the pooled predator diets, and Schoener's index (*α*; Equation (2); Schoener, 1970) was calculated from the pooled diets of each pairwise comparison of predator species within each of the sampling periods.

(2) $$\alpha =1-0.5\times (\Sigma |p_{\mathrm{xi}}-p_{\mathrm{yi}}|)$$

where *p* is the proportion of sequences from the *i*th prey taxa (in this case, the *i*th ESU) in the pooled diets, and *x* and *y* represent different predator species. A Schoener's *α* of 0 indicates no dietary overlap, a Schoener's *α* of 1 indicates complete dietary overlap, and a value of 0.6 is typically assumed to indicate substantial biologically relevant dietary overlap (Schoener, 1970). To compare dietary overlap between the drift and postdrift periods, a permutation test was conducted. Schoener's *α* values were first paired with the Schoener's *α* value for the same pairwise species comparison from the other period, and the mean of the paired differences between the two periods was calculated as the observed value. The permutation test distribution was created by randomly inverting the assigned sampling periods for each pair of Schoener's *α* values. For each permutation, each pair of Schoener's *α* values comparing the dietary overlap of the same two predators species had an equal chance to retain the same order, or be reversed (i.e., the postdrift value was now treated as the drift period value and vice‐versa). The mean difference of the paired values was then calculated using the same method used to calculate the observed mean difference value. This process was iterated 99,999 times to generate a distribution. The observed mean difference of paired Schoener's *α* was compared to the distribution of permutation mean difference values to estimate a *p*‐value and determine significance.

Selectivity was analyzed using Chesson's selectivity index (*ε*; Equation (3); Chesson, 1983).

(3) $$\varepsilon =\left(m\times \alpha_{i}-1\right)\times {\left(\left(m-2\right)\times \alpha_{i}+1\right)}^{-1}$$

where *m* is the number of prey types in the environment and *α*_i_ is the Manly's selection index for the *i*th prey type (Equation (4); Manly, 1991).

(4) $$\alpha =\left(r_{i}/n_{i}\right)\times {\left(\Sigma r_{j}/n_{j}\right)}^{-1}$$

where *r* is the proportion of the *i*th prey taxa in the predator diet (by proportion of sequence reads in the diet sample), and *n* is the proportion of the *i*th prey item in the environment (by biomass in the drift samples). Chesson's *ε* varies on a scale from −1 to 1, with negative values indicating negative selection, and positive values indicating positive selection for a given prey item. Fourteen of the 33 ESUs detected in fish diets were captured during the drift survey, so biomass estimates and selectivity could only be calculated for these ESUs (Figure 2). Chesson's *ε* was calculated with the average diet of a predator species for each day a predator species was sampled during the electrofishing survey. A PERMANOVA was performed on Euclidean distance matrix of the daily Chesson's *ε* values, testing how predator species, sampling period, substrate, and interactions between factors affected selectivity values. If interactions were not significant, a new PERMANOVA was performed without the interactions to test for significance of factors. Principal components analysis (PCA) was performed on the Euclidean distance matrix and the first two principal coordinates were plotted.

## 3 RESULTS

### 3.1 DNA sequencing

Metabarcoding analysis of diet samples successfully amplified sequences of prey items from 287 predators collected during the electrofishing survey. Of the total fish collected, 355 GI tract samples (96.7%) successfully amplified the target 18S region. Of the samples that successfully amplified, 68 (19.4%) contained <1% of prey DNA and were removed from further analysis. Diet samples from 12 predator species (Table 3) were successfully amplified and contained prey DNA (*n* = 287).

**Table 3:** Predator species caught during electrofishing surveys and sample sizes for diet samples collected during each sampling period (“drift” during larval lake sturgeon dispersal from spawning sites from late May to June, and “post‐drift” after dispersal in early July). Sample sizes only include samples from fish that could be rarefied to 1950 sequences contained >1% of sequences from prey taxa

Predator species	Three‐letter species code	Sample size Drift, postdrift	Sample size Sand, gravel	Total
Blackside darter (*Percina maculata*)	BSD	11, 6	12, 5	17
Burbot (*Lota lota)*	BUR	9, 7	10, 6	16
Central mudminnow (*Umbra limi*)	CMM	7, 7	7, 7	14
Common shiner (*Luxilus cornutus*)	CMS	3, 2	3, 2	5
Creek chub (*Semotilus atromaculatus*)	CRC	7, 6	4, 9	13
Hornyhead chub (*Nocomis biguttatus*)	HHC	36, 13	39, 10	49
Logperch (*Percina caprodes*)	LOP	14, 7	21, 0	21
Pumpkinseed (*Lepomis gibbosus*)	PUS	8, 5	0, 13	13
Rainbow darter (*Etheostoma caeruleum*)	RAD	27, 18	28, 17	45
Rock bass (*Amblopites rupestris*)	ROB	28, 9	16, 21	37
Smallmouth bass (*Micropterus dolomieu*)	SMB	24, 2	11, 15	26
White sucker (*Catostomus commersonii*)	WHS	5, 5	5, 5	10
Yellow perch (*Perca flavescens*)	YEP	14, 7	9, 12	21
Total		192, 95	165, 122	287

**Figure 3:**
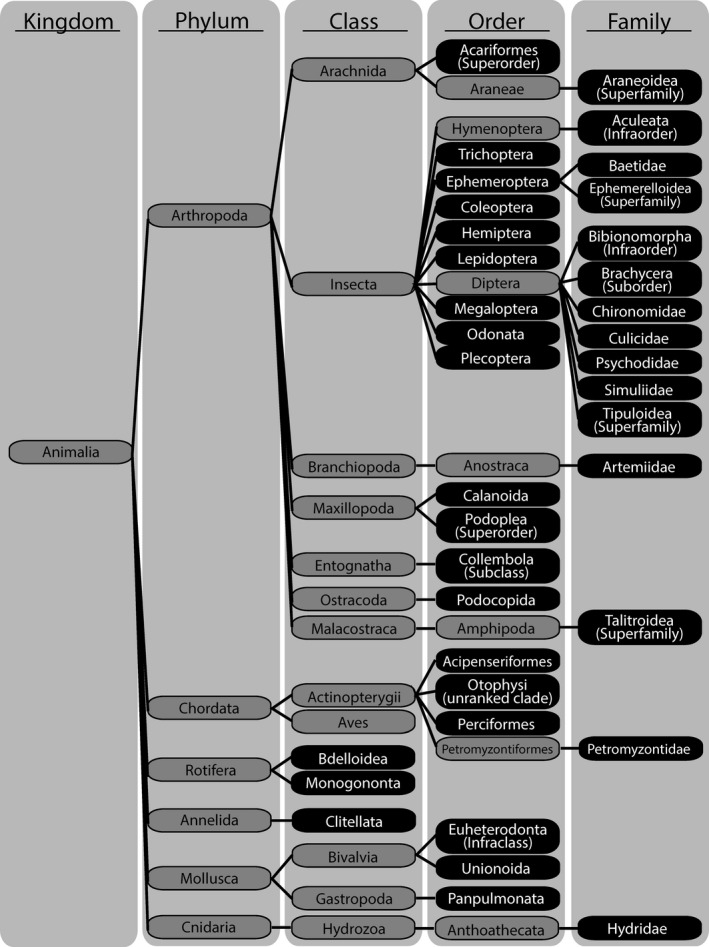
Taxonomic relationships of the prey and predator taxa identified by DNA sequencing of the 18S V9 rRNA gene in predator GI tracts. The 39 ESUs (in black) are the lowest taxonomical unit to which OTUs could be identified with >95% identity. All predators fell into the Otophysi and Perciformes clades and were subject to intraguild predation by other predator taxa

Samples rarified to 1,950 sequences contained 10,597 unique OTUs. Of the 2,500 most abundant OTUs, 375 were identified as potential prey items based on alignment to reference sequences on GenBank. Suspected predator DNA accounted for 43.05% of the total sequence reads and was removed from further analyses. Prey sequences accounted for 17.74% of all sequences from the GI tract samples. Prey sequences from these samples were grouped into 39 ESUs based on the lowest taxonomic classification that could be determined from Genbank (Figure 3). The most abundant sequences in the pool of potential prey were identified as Ephemeroptera (27.96%), Otomorpha (10.32%), and Simuliidae (10.03%). The 15 ESUs that were represented in the drift biomass data used to examine selectivity account for 76.57% of the sequence reads from the 39 prey ESUs (12.88% of total sequences).

### 3.2 Relationship between biomass and sequence abundance

Sequences of larval fish and invertebrates were recovered from all subsamples of the homogenates collected during the drift surveys. Samples rarified to 9,450 sequences contained 413 unique OTUs. Of these, 398 (96.4%) were identified as sequences from the prey items included in the homogenate based on alignment to reference sequences on GenBank. Sequences from three families of Ephemeroptera (Isonychiidae, Siphlonuridae, and Heptageniidae) could not be distinguished from each other, so these were combined into one category. Likewise, two families of Trichoptera (Leptoceridae and Hydropsychidae) could not be distinguished from each other by sequence and were combined into one category.

Sequence abundance was correlated with biomass in the sample (Pearson's correlation, *R* ^2^ = 0.389, *p* < 0.001). RCFs ranged from 3.495 to 0.009, indicating the 18S rRNA universal primers were biased toward certain taxa (notably Families: Acipenseridae, Cambaridae, and Isonychiidae/Siphlonuridae/Heptageniidae; Table 2).

### 3.3 Diet characterization

**Figure 4:**
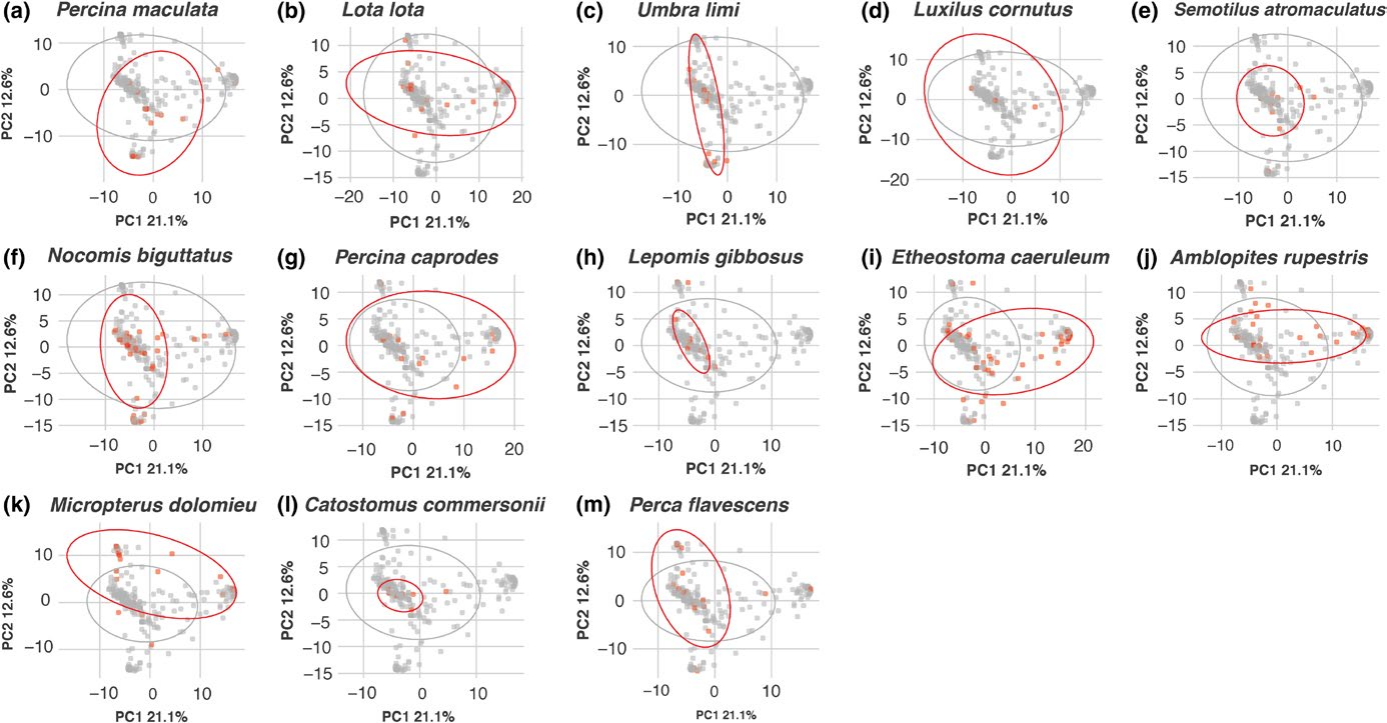
Principal coordinates analysis (PCoA) of diet compositional variation from all diet samples highlighted by each predator species compared to all other predators. Variation in PC1 is mainly associated with higher prevalence of Ephemeroptera (right) or fishes and rotifers (left) sequences in diet samples. Variation in PC2 is mainly associated with higher prevalence of Otomorpha (top) or Simuliidae (bottom) sequences in diet samples. Each diet sample is represented by a point and color identifying predator species, with the species named in the title above each plot in red and all others in gray

Predator diets contained between 1 and 10 diet items from different ESUs (mean = 3.8). The average proportion of reads from each ESU was calculated for each species of predator (Table 4). PCoA of the diet proportions revealed that diets segregated mainly by prevalence of a handful of prey items (Figure 4). High prevalence of mayflies versus fish and rotifers was the most important prey taxa contributing to the variation in diet composition across all species, explaining 21.1% of the variation (PC1; Figure 4). The second most important distinction in diet composition was between diets that contained more otomorph fishes (encompassing the cyprinids and catostomids observed in this study) compared to diets which contained more simuliid fly sequences (PC2; Figure 4).

**Table 4:** Proportion of the DNA reads from all ecologically significant units (ESUs) observed in the diets of each predator species after suspected predator sequences had been removed

	Predator species												
	*Percina maculata*	*Percina caprodes*	*Etheosotma caeruleum*	*Perca flavescens*	*Lepomis gibbosus*	*Micropterus dolomieu*	*Ambloplites rupestris*	*Nocomis biguttatus*	*Semotilus atromaculatus*	*Luxilus cornutus*	*Umbra limi*	*Lota lota*	*Catostomus commersonii*
Prey taxa													
Vertebrates													
Perciformes	—	—	—	—	—	—	—	0.14	0.42	0.01	0.09	0.22	0.26
Otomorpha	0.07	0.15	0.05	0.25	0.17	0.36	0.13	—	—	—	0.02	0.09	—
Acipenseriformes	—	0.05	0.03	0.09	0.01	0.04	0.02	0.07	0.02	—	0.07	0.04	—
Petromyzontiformes	—	—	—	—	—	—	—	—	—	—	——	—	—
Aves	—	—	—	—	—	—	—	0.01	—	—	—	—	
Mayflies													
Baetidae	0.42	0.02	0.11	0.01	—	0.02	0.05	0.01	0.03	0.01	—	0.03	—
Ephemerelloidea	0.02	0.08	0.04	—	—	0.01	—	—	0.01	—	—	—	—
Other Ephemeroptera	0.10	0.28	0.32	0.11	0.01	0.27	0.23	0.07	0.05	0.05	0.01	0.16	0.03
Aquatic insects													
Coleoptera	—	0.01	—	—	—	—	—	0.02	—	—	0.05	—	—
Lepidoptera	—	—	—	—	—	—	0.01	—	—	0.06	—	—	—
Megaloptera	—	—	—	0.02	—	—	—	0.01	—	—	—	—	—
Odonata	—	—	—	—	0.04	—	0.02	—	0.01		—	—	—
Plecoptera	—	—	—	0.01	—	—	0.04	0.02	—	—	—	0.09	—
Trichoptera	0.02	0.05	0.08	0.05	—	0.04	0.02	0.05	0.02	—	0.01	0.07	0.01
Flies and midges													
Bibionomorpha	—	—	—	—	—	—	—	0.02	—	—	—	—	—
Brachycera	—	—	0.01	0.01	—	0.04	0.03	—	0.04	—	—	—	—
Chironomidae	0.05	0.10	0.11	0.04	0.13	0.03	0.10	0.03	0.04	—	0.02	0.02	0.09
Culicidae	—	—	—	—	—	—	—	—	—	—	—	—	—
Psychodidae	—	—	—	—	—	—	0.01	—	—	—	—	—	—
Simuliidae	0.19	0.10	0.09	0.10	0.01	0.02	—	0.16	0.08	0.22	0.24	0.03	0.10
Tipuloidea	—	0.03	—	—	—	—	0.01	—	0.06	—	—	—	—
Terrestrial Arthropods													
Acariformes	—	—	0.04	0.03	—	—	0.02	0.04	0.09	0.25	0.01	0.04	—
Araneoidea	—	—	—	—	—	—	—	0.02	—	—	—	—	—
Hemiptera	—	—	—	—	—	—	—	—	—	—	—	—	—
Aculeata	—	—	—	0.01	—	—	—	0.01	0.03	—	—	—	—
Collembola	—	0.04	0.01	—	—	—	—	0.01	—	—	0.01	0.01	—
Crustaceans													
Artemiidae	—	—	—	—	0.01	—	0.01	—	0.01	—	—	—	—
Calanoida	—	—	0	—	—	—	—	—	—	—	—	—	—
Podocopida	—	—	—	0.05	0.05	0.04	0.03	0.02	—	—	0.02	—	0.31
Podoplea	—	0.02	—	0.05	—	—	0.03	—	—	0.20	0.13	0.05	0.05
Talitroidea	—	—	—		—	—	—	—	—	—	0.05	—	—
Clitellata (Worms)	—	—	0.02	0.06	0.04	0.04	0.10	0.02	—	—	0.07	—	—
Mollusks													
Euheterodonta	—	—	—	—	0.15	—	—	—	—	—	0.07	—	0.10
Panpulmonata	—	—	—	—	0.23	—	0.01	—	—	—	0.07	—	—
Unionoida	0.05	—	—	0.03	—	—	—	—	—	—	—	—	—
Metazoans													
Bdelloidea	—	0.03	0.07	0.04	0.10	0.04	0.06	0.10	—	0.13	0.05	0.02	0.02
Eutardigrada	—	—	—	0.05	0.01	—	0.02	0.05	—	—	—	—	0.01
Hydra	—	—	—		—	0.01	—	—	—	—	—	—	—
Monogononta	0.05	0.03	0.01	0.02	0.04	0.03	0.04	0.11	0.07	—	0.01	0.14	—

**Figure 5:**
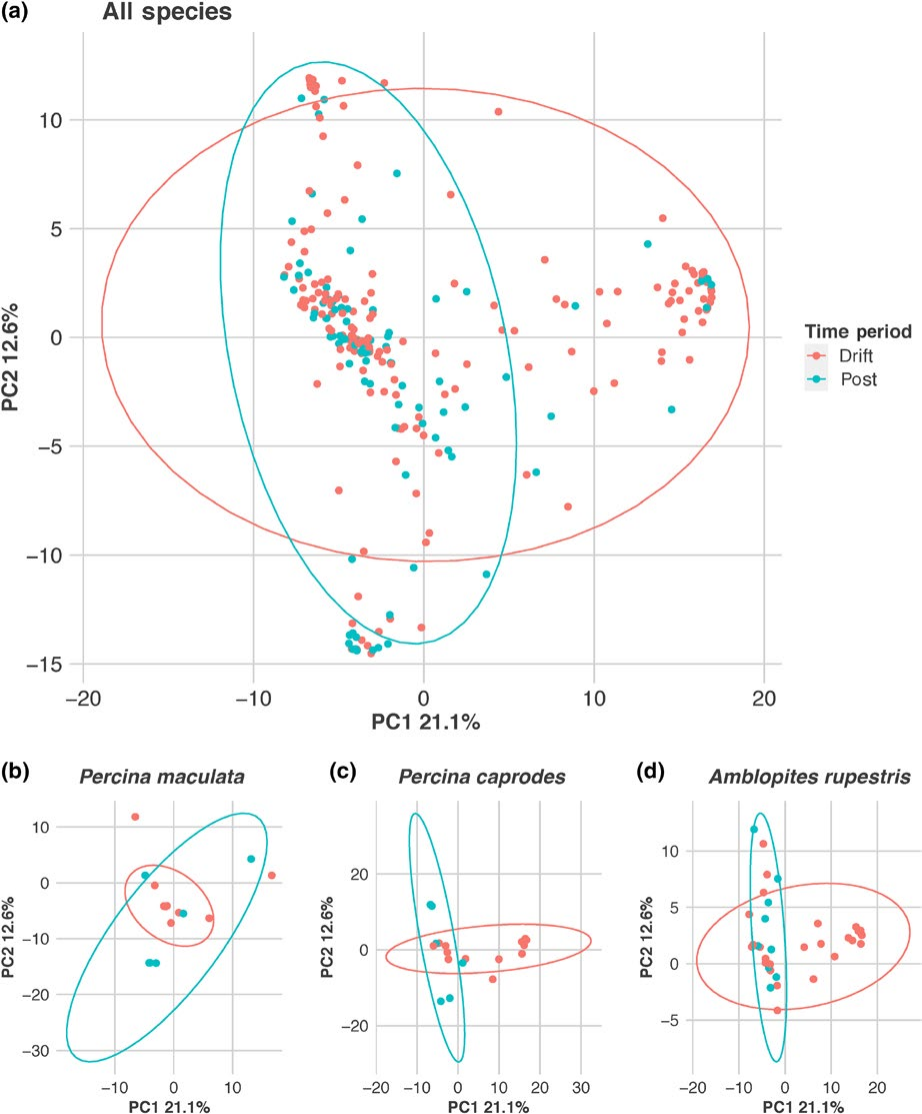
Principal coordinates analysis (PCoA) of diet taxonomic compositional variation from all diet samples highlighted by time period. PC1 is mainly associated with higher prevalence of Ephemeroptera (right) or fishes and rotifers (left) sequences in diet samples. PC2 is mainly associated with higher prevalence of Otomorpha (top) or Simuliidae (bottom) sequences in diet samples. PCoA results are presented for all samples (a) and for the three predator species with significant changes in diet between the drift and postdrift periods (b–d). Each diet sample is represented by a point and color identifying sampling period

PERMANOVA results indicated that there was no significant three‐way interaction between substrate, sampling period, and predator species in influencing predator diets (pseudo‐*F* = 1.04, *p* = 0.351). With the three‐way interaction removed, there were significant interactions between the predator species and sampling period (pseudo‐*F* = 1.46, *p* = 0.001; Figure 5a) and between substrate and sampling period (pseudo‐*F* = 2.22, *p* = 0.013; Table 5). PERMANOVA results for separate predator species testing the effect of sampling period on diet composition revealed that three predator species had significantly different diets in the two sampling periods after Holm–Bonferroni correction; blackside darter [*Percina maculata *(Girard, 1859); pseudo‐*F* = 5.02, *p* = 0.001], logperch [*Percina caprodes *(Rafinesque, 1818); pseuso‐*F* = 3.89, *p* = 0.001], and rock bass [*Ambloplites rupestris *(Rafinesque, 1817); pseudo‐*F* = 3.24, *p* = 0.002; Table 6; Figures 5b–d].

**Table 5:** Results of PERMANOVA analysis quantifying effects of predator species (*n* = 13), substrate (sand or gravel), and time period (during or after drift) on the prey composition of diet samples. Tests reveal that the diet compositions of predator species and predators sampled in areas of the river characterized by different substrates differed between different time periods

Factor	*df*	Pseudo‐*F*	*R* ^2^	*p*‐Value
Predator species	12	3.274	0.120	0.001*
Substrate	1	3.601	0.011	0.001*
Time period	1	4.269	0.013	0.001*
Predator:Substrate	10	1.022	0.031	0.388
Substrate:Time	1	2.225	0.007	0.013*
Predator:Time	12	1.456	0.053	0.001*
Residual	249		0.764	

^*^ Result was statistically significant (*p* < 0.05).

**Table 6:** PERMANOVA analysis results quantifying the effect of time period (during or after the period of high drift biomass) on diet composition of each predator species

Predator species	*p*‐Value
Blackside darter (*Percina maculata*)	0.001^a^
Burbot (*Lota lota)*	0.047
Central mudminnow (*Umbra limi*)	0.516
Common shiner (*Luxilus cornutus*)	1
Creek chub (*Semotilus atromaculatus*)	0.707
Hornyhead chub (*Nocomis biguttatus*)	0.647
Logperch (*Percina caprodes*)	0.001^a^
Pumpkinseed (*Lepomis gibbosus*)	0.520
Rainbow darter (*Etheostoma caeruleum*)	0.009
Rock bass (*Amblopites rupestris*)	0.002^a^
Smallmouth bass (*Micropterus dolomieu*)	0.989
White sucker (*Catostomus commersonii*)	0.522
Yellow perch (*Perca flavescens*)	0.658

a Diet compositions of predator species that were significantly different between the two time periods after Bonferroni correction for multiple comparisons (*α* = 0.004).

### 3.4 Dietary overlap

Schoener's index of dietary overlap (*α*) was calculated for each pair of predator species during each time period (Table 7). Five species pairs had substantial dietary overlap during the drift period: rainbow darter (*Etheosotma caeruleum* Storer, 1845) and logperch (*α* = 0.79), rainbow darter and rock bass (*α* = 0.65), rainbow darter and smallmouth bass (*Micropterus dolomieu *Lacepède, 1802; *α* = 0.61), rock bass and smallmouth bass (*α* = 0.61), and smallmouth bass and yellow perch [*Perca flavescens *(Mitchill, 1814); *α* = 0.63]. There were no species pairs with substantial dietary overlap during the postdrift period. A permutation test of the Schoener's *α* values for each species pair shows that overall, dietary overlap slightly but significantly decreased from the drift period in late May/early June to the postdrift period in early July (mean Δ*α* = −0.05, *p* = 0.003). This change can mostly be attributed to decrease in overlap between the species pairs with the highest overlap during the drift period, with the largest changes coming from rainbow darter and logperch (Δ*α* = −0.435) and smallmouth bass and rainbow darter (Δ*α* = −0.39).

**Table 7:** Schoener's index of dietary overlap (*α*) between each pair of predator fish species represented in the data set

	BSD	BUR	CMM	CMS	CRC	HHC	LOP	PUS	RAD	ROB	SMB	WHS
BUR	0.30 (0.14)											
CMM	0.11 (0.19)	0.31 (0.31)										
CMS	0.04 (0.22)	0.19 (0.14)	0.48 (0.19)									
CRC	0.27 (0.23)	0.41 (0.49)	0.16 (0.20)	0.23 (0.20)								
HHC	0.25 (0.36)	0.53 (0.35)	0.36 (0.50)	0.19 (0.24)	0.44 (0.49)							
LOP	0.24 (0.36)	0.46 (0.27)	0.18 (0.24)	0.05 (0.16)	0.26 (0.24)	0.38 (0.38)						
PUS	0.17 (0.12)	0.10 (0.09)	0.28 (0.20)	0.01 (0.07)	0.11 (0.10)	0.27 (0.10)	0.10 (0.23)					
RAD	0.37 (0.42)	0.51 (0.23)	0.23 (0.26)	0.06 (0.37)	0.29 (0.23)	0.44 (0.25)	0.79^a^ (0.36)	0.23 (0.26)				
ROB	0.33 (0.15)	0.55 (0.33)	0.30 (0.15)	0.05 (0.22)	0.26 (0.16)	0.38 (0.18)	0.49 (0.49)	0.32 (0.39)	0.65^a^ (0.35)			
SMB	0.33 (0.16)	0.45 (0.21)	0.23 (0.04)	0.03 (0.13)	0.22 (0.06)	0.38 (0.02)	0.47 (0.39)	0.45 (0.06)	0.61^a^ (0.21)	0.61^a^ (0.40)		
WHS	0.12 (0.07)	0.33 (0.39)	0.43 (0.31)	0.30 (0.05)	0.32 (0.40)	0.40 (0.28)	0.15 (0.17)	0.11 (0.42)	0.18 (0.22)	0.17 (0.32)	0.17 (0.14)	
YEP	0.25 (0.28)	0.28 (0.26)	0.36 (0.36)	0.17 (0.25)	0.20 (0.17)	0.51 (0.22)	0.27 (0.34)	0.45 (0.19)	0.41 (0.43)	0.36 (0.36)	0.63^a^ (0.36)	0.32 (0.17)

Note

Top values represent dietary overlap during larval lake sturgeon drift, and the bottom values in parentheses indicate dietary overlap during the postdrift sampling period. Schoener's *α* values >0.6 are considered to be biologically relevant and indicate the possibility of competition. See Table 3 for three‐letter predator fish codes.

a Predator species with substantial dietary overlap (Schoener's *α* > 0.60).

### 3.5 Prey availability and diet selectivity

The proportions of total drift biomass and the proportions of total reads in the predator GI tract samples for 15 prey ESUs were estimated for each night (Table 8). On average, prey biomass was higher during the drift period than during the postdrift period (Figure 2; Table 1). Catostomid larvae and mayflies in the “Other Ephemeroptera” ESU (primarily the family Isonychiidae) were the most abundant prey by biomass during the drift period (mean nightly catch dry weight biomasses of 11.69 and 2.12 g respectively). During the postdrift period, Trichoptera and Plecoptera became the most abundant prey taxa (mean nightly catch dry weight biomasses of 1.09 and 1.66 g, respectively). Mean biomass of Trichoptera and Plecoptera did not change substantially from drift to postdrift periods; however, biomass of other prey taxa declined, largely due to emergence of several mayfly families throughout June.

**Table 8:** Percentages of total estimated drift biomass (top) and percentages of reads in predator diet samples (bottom) for the 15 prey ecologically significant units (ESUs) morphologically identified from drift survey samples for each night of the drift survey

	Prey ESUs														
	Otomorpha	Acipenseriformes	Hemiptera	Baetidae	Ephemerelloidea	Other Ephemeroptera	Trichoptera	Plecoptera	Chironomidae	Simuliidae	Tipuloidea	Megaloptera	Talitroidea	Coleoptera	Odonata
Drift sampling date															
23/5/2015	46.68 (1.43)	9.17 (3.22)	—	— (9.54)	0.83 (3.39)	22.22 (39.44)	7.34 (3.85)	7.37 (1.31)	0.07 (4.18)	— (7.42)	— (2.22)	— (0.11)	0.07 (—)	0.91 (—)	5.33 (0.90)
4/6/2015	19.94 (10.29)	36.11 (5.91)	—	0.11 (4.41)	1.16 (3.52)	25.02 (16.47)	8.81 (4.98)	4.24 (0.45)	0.28 (2.48)	— (14.78)	1.53 (—)	—	0.18 (—)	2.61 (0.22)	—
5/6/2015	32.03 (8.68)	20.05 (6.21)	—	0.20 (6.56)	0.68 (1.54)	10.88 (18.49)	21.04 (2.17)	6.03 (4.06)	0.39 (4.74)	— (4.73)	— (1.69)	— (0.03)	0.07 (—)	2.89 (—)	5.81 (—)
6/6/2015	88.37 (21.41)	1.58 (—)	0.04 (—)	0.12 (7.30)	0.42 (0.08)	1.90 (19.41)	2.11 (5.29)	2.66 (—)	0.25 (6.73)	— (4.04)	—	0.15 (—)	0.03 (—)	0.82 (—)	1.54 (0.52)
7/6/2015	49.08 (18.40)	0.74 (5.40)	— (0.06)	0.05 (8.98)	0.78 (0.53)	5.58 (4.55)	4.82 (1.96)	24.73 (0.34)	— (2.23)	— (3.97)	—	—	—	0.13 (0.06)	13.94 (2.48)
3/7/2015	17.95 (15.65)	— (6.21)	—	3.74 (1.58)	— (2.15)	13.66 (8.86)	23.83 (2.57)	29.68 (3.74)	0.65 (10.89)	0.11 (10.95)	— (0.11)	—	—	1.77 (5.45)	8.62 (—)
5/7/2015	14.38 (6.09)	— (5.02)	—	1.13 (2.60)	0.43 (—)	19.12 (10.42)	22.31 (5.92)	38.04 (—)	0.37 (11.46)	0.21 (11.14)	— (0.01)	—	0.20 (2.63)	3.79 (—)	—

**Figure 6:**
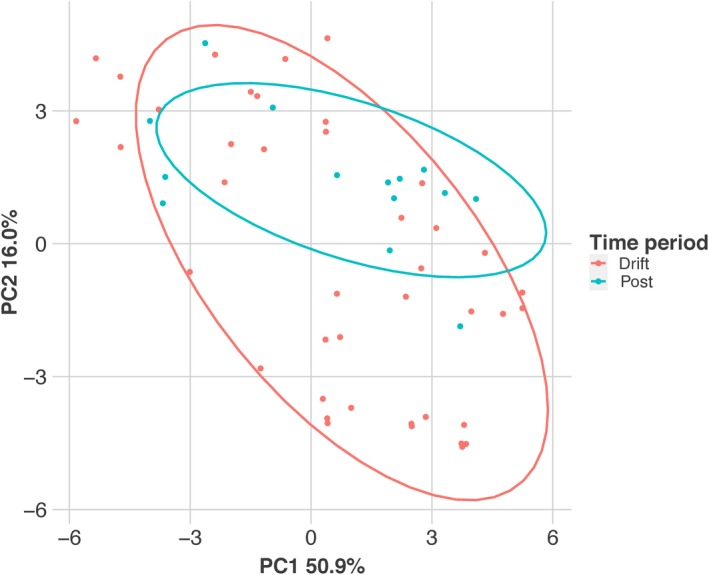
Principal components analysis (PCA) characterizing variation predator diet preference with each point representing the pooled diets from fish of the same species sampled on the same day. PC1 is mainly associated with the higher preference for Chironomidae (left), or Coleoptera, Plecoptera, and Trichoptera (right). PC2 is mainly associated with higher preference for Baetidae (top) or other Ephemeropterans (bottom) sequences in diet samples. Each diet sample is represented by a point and color identifying sampling period

Chesson's selectivity index value (*ε*) was calculated for each prey ESU by pooling reads by predator species for each day of sampling. PERMANOVA of the *ε* values indicate that there was only a significant interaction between sampling period and predator species affecting selectivity of prey items. PCA of the Chesson's *ε* distance matrix revealed diet preferences were largely consistent between periods, as there was no obvious difference in the distribution of predator references along the first PCA axis that explained most of the observed variation (PC1, Figure 6). There did seem to be a shift in some predator preferences toward baetid mayflies and away from other mayflies during the postdrift period (PC2, Figure 6).

## 4 DISCUSSION

Metabarcoding of the 18S V9 region of rRNA combined with field surveys of the prey community allowed quantification of changes in predator diets as the availability and taxonomic composition of prey changed. The taxonomic makeup of diets as characterized by metabarcoding analyses were largely concordant with the current knowledgebase for the diets of the predator species sampled in this study. Sequencing analysis revealed that many of the riverine fish predators analyzed in this study had diverse diets, with nine or more prey taxa identified at least to the class level contributing at least 1% of the prey sequences within the diet samples of each predator species. This high diversity observed in this study can largely be attributed to the ability of metabarcoding to detect quickly digested soft‐bodied prey that are often difficult to identify in morphological analyses of diet samples (Albaina et al., 2016; Alonso et al., 2014; Moran, Orth, Schitt, Hallerman, & Aguilar, 2016; Sakaguchi et al., 2017).

Combining the dietary composition data with information on the abundance of prey taxa provided insight into how changes in the prey community affected the dietary patterns of predators. There was a significant shift in the diet composition of the predator community as a whole as the overall biomass of the prey community decreased and the relative abundances of prey taxa changed between drift and postdrift periods. Dietary overlap between predator species decreased as prey decreased in abundance, possibly due to niche partitioning to avoid intense competition for less abundant prey resources. Finally, while dietary composition changed, predator preferences remained stable. Predator preferences were not dependent on the availability of prey in the environment. High biomass of a few prey taxa preferred by many predators seemed to drive the relatively higher diet overlap during the drift period. As these preferred prey taxa declined in abundance and the prey community was not dominated by a few taxa, differences in predator preferences drove the reduction in diet overlap seen in the postdrift period. Including surveys of prey communities with diet analysis can greatly improve metabarcoding diets studies, both from a technical stance by enabling researchers to test for bias in their molecular assays, but also by providing a greater ecological context to interpret diet composition and dietary shifts.

### 4.1 Diet characterization

While diets of riverine fish predators were more diverse based on metabarcoding analysis than morphological diet analysis of these species has previously recorded, the identities of the most prevalent prey items were largely consistent with previous dietary observations of the particular predator species in this study. Three darter species sampled in the UBR (logperch, blackside darter, and rainbow darter) preyed primarily on mayfly (ESUs: Baetidae, Ephemerelloidea, and Other Ephemeroptera) and midge larvae (Families: Chironomidae and Simuliidae), which is similar to the findings of previous studies (Alford & Beckett, 2007; Phillips & Kilambi, 1996). Pumpkinseed sunfish [*Lepomis gibbosus *(Linnaeus, 1758)] appeared to specialize on mollusks, snails (Order: Panpulmonata) and clams (Infraclass: Euheterodonta), along with smaller contributions to the diet from other benthic invertebrates and cyprinids, all of which have been observed in other systems (García‐Berthou & Moreno‐Amich, 2000; Locke, Bulté, Forbes, & Marcogliese, 2013; Mittelbach, 1984). Most of the yellow perch [*Perca flavescens *(Mitchill, 1814)] collected during the electrofishing survey appeared to be age‐1 fish. Metabarcoding results for these young yellow perch support previous findings that this life stage has a diverse diet, and larval fishes are a particularly important prey for juvenile yellow perch (Parke, Uzarski, Ruetz III, & Burton, 2009). Diets of minnows and chubs (Family: Cyprinidae) were quite diverse, but largely characterized by small dipteran larvae, consistent with past studies (Johnson, 2015; Quist, Bower, & Hubert, 2006). Central mudminnow [*Umbra limi *(Kirtland, 1841)] exhibited a diet similar to other analyses that focused primarily on midge larvae crustacean zooplankton, and mollusks (Chilton, Martin, & Gee, 1984; Martin‐Bergmann & Gee, 1985). White sucker were shown to prey largely on ostracod crustaceans, a common prey item observed in other studies (Ahlgren, 1990), but the molecular diet analysis also revealed that white sucker in the UBR may also engage in piscivory or consume the eggs and larvae of spawning bass, perch, and darters, which has also been observed in other studies (Baldridge & Lodge, 2013).

The large‐bodied predators of the UBR had somewhat similar diets largely consisting of other fishes (ESUs: Perciformes [burbot only], Otomorpha, Acipenseriformes), and some of the larger macroinvertebrates (ESUs: Other Ephemeroptera, Plecoptera, Trichoptera, Clitellata). There was a wide range of size classes of burbot, rock bass, and smallmouth bass present in the UBR (total lengths ranging from 75 to 255 mm, 41 to 310 mm, and 59 to 508 mm, respectively). The effect of size class on diet composition was not analyzed in this study, so findings do not take into account ontogenetic diet shifts that may occur in these species (Amundsen et al., 2003; Dauwalter & Fisher, 2008; Paterson, Drouillard, & Haffner, 2006) that could explain dietary breadth. The smaller size classes of these species may prey heavily on aquatic macroinvertebrates while larger fish account for most of the piscivory seen in the data from this study (Amundsen et al., 2003; Dauwalter & Fisher, 2008; Paterson et al., 2006). As a result, the diets of some size classes may overlap more strongly with other species. Additionally, cannibalism and consumption of other Perciformes are a component in the diets of rock bass and smallmouth bass (Clady, 1974; Frey, Bozek, Edwards, & Newman, 2003) but could not be detected due to the low taxonomic resolution of the 18S V9 region of rRNA. Using markers for a more variable region in fishes (e.g., mitochondrial cytochrome oxidase I) would improve the ability to detect fishes in the diets of close relatives (Trebitz, Hoffman, Grant, Billehus, & Pilgrim, 2015). However, use of multiple primers that optimize classification potential of different groups would preclude our ability to estimate relative sequence abundance (and thus inferentially relative biomass) across all prey taxa.

Notably, crayfish (Family: Cambaridae) were absent from the OTUs included in the diet analysis despite the test drift homogenate samples indicating that the 18S rRNA universal primers had a substantial predisposition to overrepresent crayfish DNA. Crayfish were important components of diets in some of the species present in the UBR according to other studies (Dauwalter & Fisher, 2008; Paterson et al., 2006). Adult crayfish were present in the UBR during the entire sampling period, but most predator fish sampled for this study would have been too small to prey on adult crayfish. Juvenile crayfish were a significant part of the drift in mid‐late June (data not shown), but numbers had dramatically decreased by July when sampling during the postdrift period occurred.

### 4.2 Dietary overlap, niche partitioning, and predator preferences

All prey taxa were consumed by multiple predators; however, there were relatively few pairwise predator comparisons showing substantial dietary overlap (Schoener's *α* > 0.60). Overall, dietary overlap among all species was typically higher during the drift period when prey was more abundant. It was only during the drift period that any predators appeared to have substantial dietary overlap (Schoener's *α* > 0.60; Table 7). High dietary overlap during the drift period does not necessarily indicate that predators are experiencing negative effects due to intense competition (Cardona, 2001; Jacobs, Madenjian, Bunnell, & Holuszko, 2010; Raborn, Miranda, & Driscoll, 2004). Niche theory actually predicts that the high abundance of prey reduces interspecific competition pressure, allowing predators to utilize the same resources (Pianka, 1974, 1976; Schoener, 1974). This pattern has also been observed in other fish communities (Correa & Winemiller, 2014; Dantas et al., 2013; Gray et al., 1997; Michaletz, 1997; Sánchez‐Hernández, Gabler, & Amundsen, 2017). No predator species exhibited a high degree of dietary overlap during the postdrift period, possibly indicating niche partitioning among the predator species when prey became relatively scarce (Gray et al., 1997; Raborn et al., 2004).

Niche partitioning as prey becomes scarce would explain the patterns seen in the predators that exhibit the greatest shifts in diet between the drift and postdrift periods. Blackside darter, logperch, and rock bass had significantly altered diets between the two periods (Figures 5b–d). These three species, along with rainbow darter all relied heavily on abundant mayfly larvae during the drift period, with Ephemeropterans making up >50% of the prey sequences from the darter species and >35% of the of the prey sequences in rock bass from this period. The biomass of mayflies in the drift decreased dramatically from the drift to postdrift periods, with the average postdrift catch biomass of mayfly larvae being just 40% of the average catch biomass during drift. Consequently, these species shifted diets to include prey that exhibited more stable abundances. Blackside darter diets postdrift were composed primarily of simuliid larvae (50% of prey sequences) and mussels (Order: Unionoida; 15% of prey sequences). Rock bass diets shifted to a more piscivorous diet, as the proportion of prey sequences made up by otomorphid fishes increased by 20%. Logperch also shifted toward piscivory (ESU: Otomorpha; 43% of prey sequences), whether targeting catostomid larvae or the eggs of summer spawning fishes, as well as chironomid fly larvae (16% of prey sequences). Rainbow darter exhibited a less dramatic shift in diet, with mayfly larvae still making up 35% of the prey sequences in their postdrift period diet. While mayfly remained some of the most abundant potential prey taxa in the drift, greater competition for mayfly prey between these species could have driven the divergences seen in the postdrift period.

Predator preferences for certain prey items were not significantly different between the drift and postdrift periods (Table 9). Most of the variation in predator preferences was associated with preferences for small‐bodied fly larvae (Families: Chironomidae, Simuliidae) or larger macroinvertebrates (Orders: Coleoptera, Plecoptera, Trichoptera; PC1, Figure 6). Biomass for these groups remained stable or slightly declined in the postdrift period. The largest change in predator preferences came from reduction in preferences for nonbaetid Ephemeropterans (PC2, Figure 6), which faced a much steeper decline in biomass, to preference for baetid mayflies, which contributed more biomass to the drift during the postdrift period (Table 1). This would match the pattern seen with the predators with the greatest diet shifts. Predators compensated for falling levels of preferred mayfly prey by reducing the amount of mayfly larvae in their diets and shifting to other prey items to reflect the availability of prey in the environment. The ability for the predators to track prey availability and switch between various food sources may serve to make the overall predator community more stable (Saavedra, Rohr, Fortuna, Selva, & Bascompte, 2016).

**Table 9:** Results of PERMANOVA analysis testing effects of predator species (*n* = 13), substrate (sand or gravel), and time period (during or after drift) on the predator preferences for prey observed in the drift

Factor	*df*	*F*	*R* ^2^	*p*‐Value
Predator Species	12	1.588	0.318	0.225
Time Period	1	4.749	0.079	0.061
Substrate	1	−3.949	−0.066	0.994
Residual	40		0.764	

### 4.3 Additional considerations

Analyses in this study were conducted assuming that the number of sequence reads in a diet sample was proportional to prey OTU biomass. Evidence from other studies suggest that the number of sequencing reads is generally a good approximation of the relative biomass of organisms in a sample (Clarke, Beard, Swadling, & Deagle, 2017; Elbrecht & Leese, 2015; Evans et al., 2016; Hänfling et al., 2016), including with the same set of universal primers used in this study (Albaina et al., 2016). However, the relationship between biomass and number of sequence reads can be highly variable among taxa due to amplification bias of the primers (Albaina et al., 2016; Elbrecht & Leese, 2015). Amplification biases are heavily dependent on the primers and prey taxa in a study, and this study showed that the sequencing procedure consistently overrepresented some taxa (e.g., Family: Cambaridae) and under‐represented others (e.g., Family: Perlidae) relative to biomass. Additionally, taxonomic resolution of the prey items could be further improved through the use of different sets of barcoding primers targeting different regions (Albaina et al., 2016; Hänfling et al., 2016).

Although metabarcoding can detect a wide array of prey items, there are some drawbacks. For example, incidental consumption of environmental DNA in the water by predators and secondary predation (detection of prey of prey) can be mistaken as predation on some prey taxa (King et al., 2008; Pompanaon et al., 2012). It can be difficult to determine whether fish are actually targeting some prey items (e.g., rotifers) or whether the sequences from those taxa are showing up in fish diets because aquatic insects or other prey items were consuming certain prey taxa. Furthermore, because metabarcoding relies on unique sequences to detect prey items, prey with the same DNA sequence as the predator cannot be distinguished from predator sequences (King et al., 2008). Therefore, incidence of cannibalism or predation on related species was not represented in predator diets, which could particularly affect estimates of rock bass and smallmouth bass diets in this study. Bass were observed to prey upon darters, and likely prey on other centrarchids (Dauwalter & Fisher, 2008), but all of those fishes have indistinguishable sequences at the 18S V9 region used in this study. Using a primer targeting a more variable sequence in fishes (e.g., cytochrome oxidase I) would improve the taxonomic resolution and make a more comprehensive analysis of piscivory in these predators possible (Trebitz et al., 2015). Likewise, cannibalism has been shown to be an important component of burbot diets (Jacobs et al., 2010), but could not be detected using metabarcoding techniques employed.

The relative abundance of the prey community estimated from the drift survey could have been biased and may not have represented the true availability of prey in the UBR. D‐frame drift nets were deployed to maximize the catch of larval lake sturgeon (Auer & Baker, 2002; Smith & King, 2005), so drift surveys might have overestimated the abundance of taxa with similar benthic drifting behaviors. Prey that drifted near the surface (e.g., catostomid larvae; Corbett & Powles, 1986) are likely under‐represented in the prey community relative abundance data. Prey taxa that do not drift (e.g., Unionidae) were too small to be sampled by the 1,600 µm mesh of the D‐frame drift nets (e.g., Rotifera) or could escape from the drift nets (e.g., Perciformes) were not represented in the prey community relative abundance estimates. Only 15 of the 33 ESUs identified in this study were represented in the drift survey. As a result, the selectivity values based on the prey community composition should only be interpreted in the context of the 15 ESUs identified in the drift surveys.

## 5 CONCLUSIONS

The 18S V9 rRNA metabarcoding approach implemented in this study shows promise as a powerful tool to investigate the diets of freshwater predatory fishes, especially if combined with other primers targeting more specific groups of taxa. Diet items could be identified to similar taxonomic levels as morphological diet analyses, with the potential for metabarcoding to have even higher taxonomic resolution as more sequences and longer reads become available. Metabarcoding also revealed that predator diets were more diverse than previously thought, detecting predation on taxa such as larval fishes and rotifers that are unlikely to be accounted for using morphological diet analysis (Carreon‐Martinez et al., 2011; Hunter, Taylor, Fox, Maillard, & Taylor, 2012; Ley et al., 2014).

This study also demonstrated how fluctuating seasonal abundance of drifting aquatic insects and larval fishes can impact predatory fish diets (Correa & Winemiller, 2014; Michaletz, 1997; Raborn et al., 2004; Sánchez‐Hernández et al., 2017). High resource abundance could lead to a release from competitive pressure and reduce the niche partitioning expected under interspecific competition (Pianka, 1974). Seasonal drift serves as an important influx of energy and nutrients into riverine systems and as a competitive release for certain species, allowing them to utilize preferred prey resources without having intense resource competition from other predator species. The combination of more representative diet analysis using metabarcoding and the sampling of diets at very different periods of prey availability allow for a more complete understanding of the trophic links within complex riverine ecosystems.

The analyses conducted in this study suggest that seasonal changes in prey abundance and composition are mirrored in the diet compositions of predators and cause changes interactions between predator species. Altered flow regimes and climate change often lead to homogenization of stream habitats and lowered temporal variability, which disrupt natural macroinvertebrate and fish communities (Bunn & Arthington, 2002; MacNaughton et al., 2017; Mustonen et al., 2018) and spawning activity of fish that contribute large amounts of biomass and nutrients to river systems (Auer, 1996; Grabowski & Isely, 2007). The loss of seasonal variation in environmental conditions would likely lead to a reduction or elimination of the seasonal variation in prey community structure and abundance as seen in this study. Seasonal variation in food web structure appears to have an effect on the overall stability of riverine communities by reducing predator reliance on the presence of a certain prey resource (Saavedra et al., 2016. This study suggests seasonal variation may also be important by temporarily reducing competition between predators for preferred prey resources. Maintaining natural temporal variation and diversity of prey communities could be an important component in conservation of riverine ecosystems. More research on the prevalence and effects of seasonal food web structural variation on the resilience of riverine communities is warranted.

## CONFLICT OF INTEREST

None declared.

## AUTHOR CONTRIBUTIONS

JMW and KTS designed the study and collected samples. JMW conducted laboratory work, performed statistical analyses, and wrote the paper with input from the other authors. TLM contributed new reagents and processed DNA sequence data.

## Data Availability

FASTA sequences, datasets, and R code used to conduct the analyses in this manuscript are available on Dryad, https://doi.org/10.5061/dryad.0jm1dt2
