# Supplementary figures and images for: 18S rRNA metabarcoding diet analysis of a predatory fish community across seasonal changes in prey availability
Source: Ecol Evol. 2019 Jan 10;9(3):1410–30. doi: 10.1002/ece3.4857 (PMC6374664; doi:10.1002/ece3.4857)

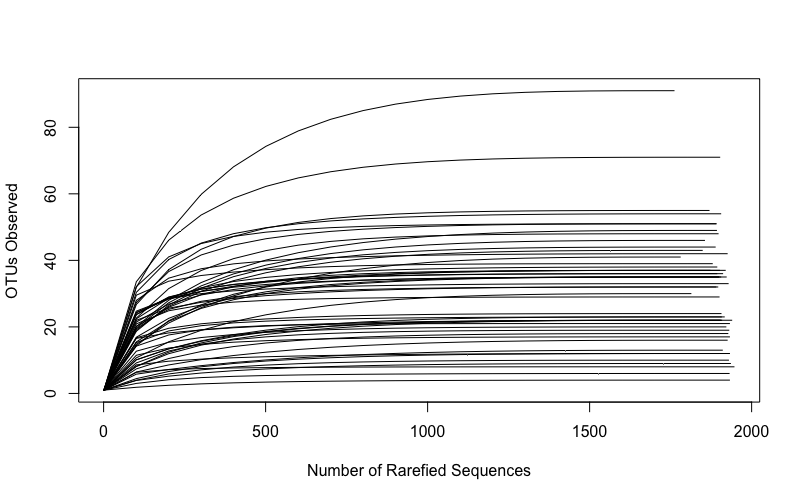

Supplement: Supplementary file 1 [file ECE3-9-1410-s001.tiff]
